# Editorial Notes

**Published:** 1906-09

**Authors:** 


### Editorial Notes.

---

**British Medical Association Meeting at Toronto.**

THE general, if not universal, opinion of those attending the Canadian meeting was one of warm admiration for the organising capabilities of the local committee, pleasurable surprise at the beauty of Toronto and its surroundings, and perhaps more especially at the handsome buildings of the University so well placed in the midst of the extensive Queen's Park. About seven hundred members attended, and these with the very fair proportion who were accompanied by wives and daughters made an appreciable addition to the town.

Those who were located in the ladies' residential colleges—some at Queen's Hall, others at Annesley Hall—within the cool, shady Queen's Park,—were fortunate, and were conveniently near the activities of the sections. The magnificent King Edward's Hotel and the Queen's, together with much private hospitality, accounted for the remainder.

Toronto may indeed be congratulated on having University buildings so commodious and well equipped, and not the least of these were the medical buildings. It was a great convenience to find that all the sections, the extensive and successful exhibition of instruments, drugs, books and appliances, the general reception rooms, ladies' rooms and secretariat, all found ample room in these University buildings, and all the more pleasant for the quiet, fresh, green park extending in every direction. The pathological museum was in a separate building, and this contained an amount of well-arranged material that must have represented months of ardent devotion to work by those responsible for its organisation.

The excessive heat caused the first few days of the Congress to be to some extent less conducive to work, but it was notable that the Canadians and Americans seemed almost more prone to suffer from the climate than the English visitors, and perhaps it made the lighter aspects of Toronto life even more enjoyable. The Royal Canadian Yacht Club's evening reception was very refreshing, on the Island half a mile out in the lake, while their hospitality in placing their steam yachts at the disposal of visitors during the week gave many cool hours for rest and reflection.

The reception by the Lieutenant-Governor of Ontario and Mrs. Mortimer Clarke, and the garden party by Mr. and Mrs. Osler in their charming residence in Toronto, as well as many other similar gatherings, showed us how pleasant both the official and purely friendly sides of Toronto society could be.

It is highly instructive to note that progressive Canada has set an example to the mother country in the development of her University system, and that in the race for material wealth the importance of higher education has not only been recognised, but has received the necessary support of well educated Canadians and of the Government to an extent that the unprovided districts of Great Britain may well emulate.

The Universities of Montreal, Toronto, Ottawa, Kingston, London, Halifax, Winnipeg and Vancouver have been launched with no meagre hand, if those institutions which it was our good fortune to inspect are fair examples of those in the other districts of the colony.

The Norman Gothic buildings of Toronto University have with good cause been praised by all visitors as unexcelled by any educational institution in the world. Of the neighbouring halls and buildings, the largest is the new department for Mineralogy and Geology. In other departments Applied Science is taught in the old School of Science, and near this are the Biological building of grey stone, the Medical building, and the University Library (containing about 100,000 volumes), the fine Pantheon-like building of the Convocation Hall, where the general meetings were held, and the Chemical Laboratory, another well equipped building. The new Physical Laboratory is soon to be added. The complete staff in Arts, Law, Medicine, and Applied Sciences numbers 243, and the students in these faculties number 2,533, the faculty of Medicine alone numbering 87 and the students 648. In addition there are the affiliated Colleges of Dentistry, Pharmacy, and Veterinary Science, and the Agricultural College at Guelph. There are also two large Conservatoires of Music, one of which has over 3,000 students. The hospitals of Toronto are excellent and complete.

At Montreal there are now two degree-granting Universities, namely McGill and Laval. The hospitals there, too, are very complete and well equipped. The Victoria Hospital with 250 beds was built and endowed by the munificence of Lord Strathcona and Mount Royal and Lord Mountstephen at an expense of over three million dollars. The Montreal General Hospital, which is shortly to be rebuilt with all latest improvements, the Victoria Hospital also with 250 beds, the Hotel Dieu, and other large medical institutions make the city in every way a well endowed one for medical teaching.

At most of the Canadian Universities a four years' course in medicine is required, but at Winnipeg the medical curriculum involves five years' study.

It is quite impossible to even attempt to do justice to the University buildings of Montreal and other cities, or to attempt to convey in a few words how successfully Canada is equipping herself in University developments. Let us hope that ere long Bristol will not be allowed to be behind these Canadian cities, of such relatively recent growth, in her efforts to keep pace with the times in having a University of her own.

**The Royal Sanitary Institute.**

The Twenty-third Congress of this Institute was held in Bristol from the 9th to the 14th of July, under the Presidency of the Right Hon. Sir Edward Fry, P.C., F.R.S., &c. A large local committee had been formed, and various executive sub-committees made ample arrangements for the reception and entertainment of a large number of visitors. These arrangements were carried out under the direction of T. J. Moss-Flower, Assoc.M.Inst.C.E., to whom much credit is due for the way in which the meeting was organised. The first meeting was at the Council House, where the members and delegates were welcomed by the Lord Mayor and the Sheriff, and the President gave an interesting and instructive inaugural address at the Victoria Rooms in the evening. A local medical journal of this kind cannot do otherwise than congratulate our city authorities on the way in which this Congress was welcomed and encouraged in its arduous work of improving the sanitary conditions of life in this country, and we are indebted to one of the Secretaries (Dr. J. R. Charles) for the following notes on some of the scientific work of the sections.

The Presidential Address of Sir William J. Collins, M.D., F.R.C.S., M.P., in the Section of Sanitary Science and Preventive Medicine, explained that the Institute exists both to promote and diffuse sanitary knowledge, and that it therefore embraces a very wide field of work. This section scrutinizes not only the environment, but also the powers within the body to do battle with disease. Witness the work of the phagocytosist and the opsonist, who attempt to exalt the powers of the blood to conquer invading germs. In 1884 he had published a paper on the question of the usually accounted specific fevers arising *de novo*, and different diseases having a common ancestry; while in 1889 he had read a paper on the further development of this thesis, maintaining that the soil as a determining factor in specificity had been absurdly underrated. He urged that the limited acceptance of the germ hypothesis is in no sense inimical to these views. The germ has been too much with us, and we have lost sight of the man amid the magnificent flora of the bacteriological laboratory. He contrasted the doctrines of the old sanitarian school with the more modern and fashionable doctrines of Pasteur and Koch. The former are imbued with the potency of such factors as go to make up environment; while the latter intent, on the microscopic causes of contagion, are bent on conferring immunity upon the individual by establishing within him a condition of insusceptibility to microscopic germs. We have expected too much from bacteriology, and have neglected the inherent properties of the cells and tissues of the body, both in the production of and protection from disease. We must go back to cellular pathology, and to cellular pathology quickened by the acceptance of the evolutionary principle. We must remember that we are "heirs of all the ages," of the lowly amœba, as well as of our parents. In inflammation, repair, and malignant growths there is a reversion to embryonal cell type. In neoplasms there is indisposition of the component cells to differentiate into tissue or suppurate; they are reminiscent of reproductive cells. Although by the agency of the leucocytes or by chemical action healthy blood is bound to be a powerful germicide, the business of the sanitarian is to prevent invasions taking place.

The subject of "The Etiology of Mediterranean Fever" was introduced in a paper by Fleet-Surgeon Bassett-Smith. A considerable amount of work has been done on the saprophytic existence of the micrococcus melitensis, and it has been proved to be an organism of great vitality. Very minute doses of the living culture were able to set up the disease by artificial inoculation in man after an incubation of five to eight days. The infection passes out of the body by the urine and alimentary canal, and infected clothing is a source of danger. Cows and goats pass out the organisms abundantly in milk and urine. The micrococcus has been found in the bodies of mosquitoes, and incubated successfully after removal from the stomach of the insect. Other possible methods of infection are through dust contaminated with infected sewage, infected food and direct inoculation.

Dr. E. Walford read a paper on the "International Notification of Disease." All the ports in the country are subject to the invasion of smallpox, and he felt that some system of international communication would be more reliable than the present methods. Under the present system notification of a case of smallpox in an English port might be the means of inflicting great loss upon an unfortunate owner of a vessel out of all proportion to the gain to anyone. A conviction for neglecting to notify is seldom possible, as the master, on whom the responsibility lies, pleads ignorance or want of diagnostic skill. A system of intercommunication between the various ports would reduce the number of these invasions. The primary object of international notification would be the concentration in a central international health office of all available information concerning the existence of epidemic diseases, and distribution of this information to the countries concerned. Dr. Fremantle, who also dealt with the subject, moved the following resolution, which was unanimously adopted:—"That this Congress do urge the Council strongly to represent to His Majesty's Government the desirability of submitting to the next Imperial Congress proposals for a system of Imperial notification of infectious disease."

Professor Bostock Hill introduced the subject of "The Health Visitor from the County Council point of view." He maintained that the lady visitor should have experience in nursing and a knowledge of hygiene. She should distribute leaflets urging the advisability of breast-feeding, and give instruction regarding the clothing and washing of infants, the importance of fresh air and the prevention of consumption.

In a paper on "Some Points in the Treatment of Outbreaks of Diphtheria," Dr. F. T. Bond explained the great difficulties under which the medical officer of health labours in dealing either with the sporadic or epidemic forms of the disease, and considered that the Public Health Acts require considerable extension and alteration, as medical science has advanced considerably since they were framed.

Dr. James Fletcher read an interesting paper on "Post-Scarlatinal Diphtheria and its Prevention." Among his methods of prevention he recommends the bacteriological examination of the throat and nose of all scarlet fever admissions, the nursing apart of all patients who are found harbouring the diphtheria bacillus, the prevention of the mingling of the convalescents from the two diseases, the bacteriological examination of nurses and maids who are transferred from the diphtheria to scarlet fever wards, and of a prophylactic dose of antitoxin to each patient in any ward in which a case of post-scarlatinal diphtheria may occur.

Dr. Fortescue-Brickdale, in his paper on "The Influence of Milk Supply on Infant Mortality," maintained that the production of a pure untreated milk was the chief point to be attained in the reform of the milk supply, especially with regard to tuberculosis and summer diarrhœa in children, of which diseases he discussed the bacteriology. He criticised the methods of boiling, prolonged heating, Pasteurisation, the addition of preservatives such as formalin, and the Budde process. He considered that we are thrown back on the only scientific method, namely that of the production of clean milk from healthy cows. This product should be kept in a sterile vessel at low temperature. The necessary subsequent modification of the milk for infant feeding should be carried out at organised milk depots.

In a paper on "Preventive Medicine and the Individual," Dr. Whitby considered that every individual should have a health-dossier, and that the money this would cost would be a good national investment. The compulsory notification of hereditarily transmissible diseases or defects is no less justifiable than that of infective fevers. The birth of the unfit cannot be checked until its inevitability under given conditions can be fully proved.

Other interesting papers in Section I. were read by Miss Marion Fitzgerald, on the "Standard of Home Life and Infant Mortality," and Mrs. Hamer Jackson, on "Prevention of Tuberculosis—A New Milk Supply."

At the Conference of Municipal Representatives, Mr. Arthur Robinson, M.P., in his paper on the "Rational Extension of Modern Cities," advocated that large tracts of prospective suburban land should be acquired by the town before it has acquired the price of building land. This land is to be laid out on plan after careful and expert consideration, but only to be built on, and then according only to the plan, when necessity arises.

Two papers dealing with "Consumption" followed. Firstly, one by Dr. Scurfield on a municipal scheme for dealing with the disease. He described the methods in use at Sheffield, where compulsory notification has been in force since 1903. On receipt of the notification a special inspector visits. He leaves a copy of instructions, advises as to the keeping of the windows open, and furnishes the poorer patients with pocket spittoons free of charge. Where the house is dirty the room is disinfected with a formalin spray. The room is also disinfected after death. Hospital and sanatorium treatment is about to be made available, while special wards in union hospitals are to some extent supplying the need for homes for advanced cases. The after-life colony is more in the future. The second paper was one by Miss Hilda Joseph on a "Labour Colony for Consumptive Convalescents." She advocated a system designed to utilise trades already learned, the work to be supplemented by gardening, poultry farming and the like. The cost of all the details of such a colony had been carefully estimated, and she did not consider that such a colony could be self-supporting for several years.

The Bishop of Hereford delivered an address on the "Hygiene of School Life," and recounted his reminiscences.

Dr. Barclay Baron dealt with the importance of the early treatment of the affections of the throat, nose and ear in children, and Dr. Symes with "Diet in Boarding Houses."

Other papers included the subject of "Hygiene in Training Colleges and in Secondary Schools."

Conferences of Medical Officers of Health, of Sanitary Inspectors, and of Women on "Hygiene" were also held; while interesting and very various discussions arose in the Sections of Engineering and Architecture, and Physics, Chemistry and Biology.

A considerable amount of attention was devoted in more than one paper to the question of dust, especially to that raised by motor-cars. Various modes of treating the roads with tar and other materials were considered at some length, but although some of them are fairly satisfactory, they are found to be prohibitive on account of their great expense. The estimate at present for a tarred road amounts to as much as £50 per mile per annum for upkeep. The use of soft stones such as limestone in the making of roads should cease in the future, and stone of a basaltic character, which is not only affected much less by wear and tear but also much less by moisture, take its place.

The meetings were well attended, and the important and interesting topics which were discussed produced many instructive debates.

**British Electro-therapeutic Society.**

On July 27th the British Electro-therapeutic Society held its provincial meeting at the Clifton Down Hotel, under the presidency of Dr. Lewis Jones. The meeting was thrown open to members of the medical profession in the district, and not a few availed themselves of the opportunity of welcoming a Society whose object is to place and maintain the medical use of electro-physical methods of therapeusis upon a scientific and professional basis.

Although it was a matter for regret that more of the members of the Society itself were unable to be present, some interesting papers were read and radiograms exhibited.

Dr. Lewis Jones, the father of the Society, gave an interesting exhibition of lantern slides prepared from radiograms of patients with cervical ribs. He dealt with the relation of these ribs to atrophy of the intrinsic muscles of the hand which occasionally is to be observed in such cases. He pointed out that the radiographic appearances of cases attended by atrophy differed in no way from those in which no atrophy could be detected.

Dr. H. Taylor (Bradford-on-Avon) read a paper on a case of rodent ulcer which had obtained benefit from zinc electrolysis; and Dr. David Arthur (London) read a paper on zinc electrolysis in general, and showed Dr. Lewis Jones's zinc surface electrodes, also an intra-uterine electrode. The chairman testified to the great usefulness of this method of treatment in cases of lupus and other disorders, but advised the use of zinc sulphate in the place of the chloride, as the former salt can be used in stronger solutions and has no caustic action.

An interesting discussion was evoked by Dr. Preston King's (Bath) paper on "Static Electricity as an Aid to Recovery." (*Vide* page 233.) The chairman considered that the reason why some patients obtained immediately a sense of well-being, while others experienced a feeling of great uneasiness while under the treatment, lay in the alteration of blood-pressure resulting from the treatment. The blood-pressure is raised, and in those persons with high pressure to start with an increase is attended by a feeling of great uneasiness, while in those of low pressure the rise is beneficial. Asthmatics are improved while under the breeze. Dr. Elliott (Chester) testified to its use in depressed conditions of the nervous system such as obtains in syphilitics.

Dr. Kenneth Wills read a comprehensive paper on "X-rays in Medical Practice," and spoke of their great value in certain skin affections, notably acne vulgaris, erythema induratum, lupus, chronic eczema, keloid, pruritus and keratosis palmaris; also of their use in certain blood diseases, and in the diagnosis of aneurysm and phthisis. He also reviewed the position of the treatment of carcinoma by means of the rays. The chairman spoke of the use of the rays in psoriasis. Dr. Shingleton Smith had seen secondary growths and nodules disappear in carcinoma under the rays, and also spoke of the promise the treatment was giving in Graves's disease. Dr. Michell Clarke testified to the use of the rays in the diagnosis of early tubercle, and in the detection of aneurysm of the descending aorta; he mentioned a case of lymphadenoma in which the rays had produced considerable benefit. Dr. Stack had found the rays of use in the diagnosis of foreign bodies in the eye, in the detection of fractures of the os magnum and splits in the bones which were mistaken for sprains, and warned against "lying radiograms." Dr. Arthur spoke of the value of a "soft" tube in the diagnosis of aneurysm.

Some interesting radiograms exhibited by Mr. J. Ellington Jones and Dr. Kenneth Wills brought this interesting meeting to a close.

**Professor of Anatomy.** THE *Journal* Committee congratulate Professor Fawcett on his distinction in having been awarded the gold medal for his M.D. thesis at the University of Edinburgh.

**Museums Association.** THE Museums Association held its seventeenth annual meeting in Bristol on July 2—5. A large attendance of curators and representatives from various British and foreign museums was welcomed by the Lord Mayor, the

Sheriff and the Museum Committee at the Council House, and the President, Dr. W. E. Hoyle, Director of the Manchester Museum, gave an interesting address on the "Education of a Museum Curator." A very successful conference was brought to a close by visits to various museums and other historic remains of the neighbourhood. Alderman Barker and his Committee must be gratified with the success which has attended their efforts on behalf of the Association and the Museums and Art Gallery of Bristol.

**Winsley Sanatorium for Consumptives.**

The first annual report of this institution was published recently. It contains much interesting information, and is in itself an excellent record of much good work already done, We now have much pleasure in adding to that report "a short survey of the first year's work," for which we are indebted to the Resident Medical Officer, Dr. E. Dunbar Townroe, who writes as follows:—

"Winsley Sanatorium was opened for the admission of patients on the 16th December, 1904. Up to that time no sanatorium existed in the three counties which aimed at the same object, namely the treatment of poorer consumptives at an early stage of the disease. In order that their object might be the better fulfilled, the Committee appointed a large number of the leading medical men in the various towns throughout the three counties of Somerset, Wilts, and Gloucester, as referees, who with the patient's medical attendant were asked to give their opinion of the case. In this way it was hoped that those cases which were eminently curable would find their way into the Sanatorium. It is not necessary here to enumerate the difficulties attending the selection of early cases. Suffice it to say that in June, 1905, six months after the Sanatorium had been opened, 75 per cent. of the cases admitted were outside the desired limit. The difficulty of obtaining suitable cases was therefore as evident at Winsley Sanatorium as at all similar philanthropic institutions. But the advantage of having a large consultative staff has been felt by the institution during the last year, until at the present date a much smaller percentage of unsuitable cases find their way to Winsley. In a small brochure published by the Committee before the opening of the institution, the hope was expressed 'that every bed would be the means of arresting the disease in two patients at least every year.' Let us see how far this hope has been fulfilled. Over 300 cases have so far been admitted to the Sanatorium. The average number of patients during the first six months was 40, whilst during the last year this has been increased to 53, there being 58 beds. Every effort has been made to keep in touch with patients after their discharge, and in this manner a fair estimate can be formed of the present condition of most of those who have left. Taking into consideration the number of *advanced* cases that found their way into the Sanatorium at first, it is not to be wondered at that twenty-two deaths should have occurred, *i.e.*, 7·3 per cent. of discharged cases; but *pari passu*, with the greater care bestowed in the selection of cases, the number arrested and returned capable of resuming work has increased in a most satisfactory manner, there being at present no fewer than 181 people who have been earning their own living for periods varying from two to ten months. It is not my purpose to discuss the after care of the consumptive working class, although this is a matter which will require most careful consideration by the public at large; suffice it to say that most attempts at procuring suitable work for discharged patients have met with success. Nowhere has this success been more marked than in Swindon, where it has been found possible, without exception, to place discharged patients in healthier surroundings without making a serious reduction in their wages. After due deliberation, the German Life Assurance Companies have come to the conclusion that sanatorium treatment for the consumptive working class is worth while. It is therefore, surely, not necessary to enter into this aspect of the question.

"As regards treatment, that which has received universal acceptance by the medical faculty as being most beneficial is adopted at Winsley Sanatorium, viz. rest, fresh air, a liberal diet, and regulated exercise under constant medical supervision.

As regards the fresh air treatment, perhaps a more admirable, equable climate could not be found in the British Isles than that of Winsley.

"With regard to food, patients have three meals a day, being expected to consume a regulated quantity of food at each meal, no attempt at the so-called stuffing diet having been made. Raw meat has formed the one unusual article of diet, each patient consuming from four to eight ounces of this article during the first month of treatment at the midday meal. Excellent results have accrued from this treatment; but in view of the known danger of tænia, this diet has not been persisted with during the summer months.

"Dealing as we are with the working classes, greater stress has been laid on the capability for work at the end of four months' treatment than on the mere gain of so many pounds avoirdupois in adipose tissue, for under conditions of rest and fresh air there is little difficulty in putting on weight, which in itself often engenders a lethargic disposition and a disinclination for exertion; whilst exercise in the form of long walks or household work is apt to become monotonous. Therefore carpentering in the woods, gardening, and outdoor engineering have been encouraged amongst the men, whilst domestic duties have been assigned the women. The greatest success has attended the patients' efforts; for whilst developing the body, the mind is healthily employed and distracted from the many family worries and cares inseparable from this class. As an instance of the utility of this form of treatment, two patients who went through a definite course of road-making whilst at the Sanatorium were certified as fit for work on discharge. They immediately resumed their former employment as platelayers on the Great Western Railway, and during the last four months they have worked from 6 a.m. to 5 p.m., Sundays included, and continue to report themselves in excellent health. In order that a true record may be kept, patients are encouraged to visit the Sanatorium once a month after discharge, and this they mostly do; thus in time it will be possible to form a true estimate of the value of treatment. But there is one aspect of the case which is of inestimable value, namely the dissemination of hygienic principles. One has but to observe the effect of sanatorium treatment on the neighbouring villages to feel confident that the educational value of such an establishment is of national importance."

### Notes on Preparations for the Sick.

